# Differences in Item Discrimination of the 25‐Question Geriatric Locomotive Function Scale Between Younger and Middle‐Aged Adults and Older Adults: An Analysis Using the Item Response Theory

**DOI:** 10.1111/ggi.70357

**Published:** 2026-02-06

**Authors:** Shunsuke Yamashina, Kenta Hirohama, Junji Nishimoto, Ryo Tanaka

**Affiliations:** ^1^ Graduate School of Humanities and Social Sciences Hiroshima University Higashihiroshima Hiroshima Japan; ^2^ Department of Environmental Medicine and Public Health, Faculty of Medicine Shimane University Izumo Shimane Japan; ^3^ Sakamidorii Hospital Hiroshima Hiroshima Japan; ^4^ Department of Rehabilitation Saitama Medical Center, Saitama Medical University Kawagoe Saitama Japan; ^5^ Department of Rehabilitation, Faculty of Health Sciences Tokyo Kasei University Sayama Saitama Japan

**Keywords:** item response theory, locomotive syndrome, screening, the 25‐question geriatric locomotive function scale

## Abstract

**Introduction:**

The 25‐item Geriatric Locomotive Function Scale is widely used to identify locomotive syndrome, a condition characterized by progressive decline in motor function. Although its reliability in older adults has been established, item‐level properties across age groups remain unclear. This study examined item discrimination and difficulty using an item response theory approach and compared item characteristics between younger and middle‐aged adults and older adults.

**Methods:**

Community‐dwelling adults aged 18 years or older were classified as younger and middle‐aged (< 65 years) or older (≥ 65 years). The graded response model estimated item discrimination and difficulty. Discrimination was interpreted using Baker's classification, while values between 0.5 and 2.5 were considered empirically acceptable. Difficulty parameters ranged approximately from −3 to +3. Model fit was assessed using the root mean square error of approximation. Wald tests compared item parameters between age groups, and sensitivity analyses used age thresholds of 60, 70, and 75 years.

**Results:**

Among 866 participants, most items showed acceptable discrimination and difficulty. Pain‐ and social participation‐related items were more discriminative in younger and middle‐aged adults, whereas mobility‐ and anxiety‐related items were more discriminative in older adults. Model fit was good (root mean square error of approximation = 0.04–0.05), and Wald tests confirmed significant item‐level differences between groups. Sensitivity analyses supported the robustness of the 65‐year cutoff.

**Conclusion:**

The Geriatric Locomotive Function Scale demonstrated statistically validated age‐related item patterns. These findings support developing age‐tailored short forms and adaptive screening tools for locomotive syndrome.

## 1 Introduction

Locomotive Syndrome (LS) is defined as a condition involving motor function deterioration and increased risk of disability, such as fractures [1]. Adults aged ≥ 65 years now comprise 29% of Japan's population [2], underscoring the need for reliable screening tools. Early detection is critical, as LS is linked to substantial physical decline within 5 years compared with individuals without LS [3]. LS is classified into non‐LS, stage 1, stage 2, and stage 3, with severe cases leading to mobility loss [4]. The prevalence of LS in Japan is 67.8% (41.3% stage 1, 14.9% stage 2, 11.6% stage 3), and 20.8% even among younger and middle‐aged adults [5, 6]. These data suggest LS can occur before older age, highlighting the need for early detection across age groups.

The 25‐question Geriatric Locomotive Function Scale (GLFS‐25) is widely used for LS screening. Its reliability and validity have been established in older adults [7, 8], and it correlates with motor function measures such as walking speed and timed up‐and‐go [9]. However, validation has largely focused on older cohorts, leaving item‐level performance in younger adults unexamined.

Each GLFS‐25 item warrants scrutiny to clarify discriminative power. The tool is effective for severe LS, but has limited ability to detect early‐stage LS [10]. This may reflect items with low discrimination or those tied to advanced functional limitation. Item discrimination indicates how well responses distinguish latent trait levels (e.g., LS severity), while difficulty indicates the latent trait level needed to endorse a response. Item response theory (IRT) enables evaluation of these parameters [11] and is recommended for assessing structural validity [12, 13]. Yet, the GLFS‐25 has not been analyzed using IRT.

Given the ordinal nature of the scale, symptoms such as pain and anxiety often appear early [14, 15], especially in younger adults [16]. In contrast, reduced activity and mobility limitations are more typical in older adults [17]. Therefore, differences in GLFS‐25 item discrimination and difficulty are expected across age groups.

This study aimed to examine the discriminative ability of each GLFS‐25 item using IRT and to compare item characteristics between younger/middle‐aged and older adults.

## 2 Materials and Methods

### 2.1 Study Design and Setting

This was a cross‐sectional study. Data were extracted from the Diagnosis, Early Detection, and Treatment of Locomotive Syndrome study using the Epidemiological Cohort “DETECt‐L” during 2021–2024. The DETECt‐L study is a community‐based cohort study designed to investigate risk factors and screening approaches related to LS syndrome. Participant recruitment was conducted through the use of flyers in Hiroshima, Kure, and Higashihiroshima City to invite voluntary participation from community‐dwelling adults. The measurements for this study were taken at local sports or community centers.

### 2.2 Participants

The inclusion criteria for this study were community‐dwelling persons aged 18 years or older with independent mobility, defined as the ability to walk outdoors without assistance from others or the use of walking aids. Participants were excluded if they were suspected of having cognitive impairment—based on self‐ or family‐reported memory loss, disorientation, or prior diagnosis of dementia—or if they had serious illnesses such as unstable cardiovascular disease, recent stroke, severe respiratory impairment, Parkinson's disease, diabetic peripheral neuropathy, or rheumatoid arthritis. Participants were divided into two age‐based groups: younger and middle‐aged adults (aged < 65 years) and older adults (aged ≥ 65 years). This classification is consistent with the thresholds commonly used in gerontological and epidemiological studies. In Japan, individuals aged 65 years and older are officially defined as older adults [18]. Participants with incomplete GLFS‐25 responses were excluded from the analysis.

### 2.3 Measurements

The measures assessed were age, sex, body mass index (BMI), and the GLFS‐25 score, which comprises 25 items (Table 1) [7]. A self‐reported evaluation was performed; each item in the GLFS‐25 was graded on a 5‐point scale from 0 to 4, and the total score was calculated. A higher score indicated greater severity of LS: ≤ 6 points indicating non‐LS; 7–15 points, LS stage 1; 16–23 points, LS stage 2; and ≥ 24 points, LS stage 3. The GLFS‐25 was self‐administered in a face‐to‐face setting at community centers. Trained staff were present to provide support as needed during questionnaire completion.

**TABLE 1:** The 25‐question geriatric locomotive function scale.

The following questions are asking about your health status and usual daily life, relating to the involvement of your back and limbs. Please answer on your state over the last one month. Please check the box for the most suitable response to each question.
• Following are questions about your body pain for the last one month:
1. Did you have any pain (including numbness) in your neck or upper limbs (shoulder, arm, or hand)?
□No pain □Mild pain □Moderate pain □Considerable pain □Severe pain
2. Did you have any pain in your back or lower back or buttocks?
□No pain □Mild pain □Moderate pain □Considerable pain □Severe pain
3. Did you have any pain (including numbness) in your lower limbs (hip, thigh, knee, calf, shin, ankle, or foot)?
□No pain □Mild pain □Moderate pain □Considerable pain □Severe pain
4. To what extent has it been painful to move your body in daily life?
□No pain □Mild pain □Moderate pain □Considerable pain □Severe pain
• Following are questions about your usual daily life for the last one month:
5. To what extent has it been difficult to get up from a bed or lie down?
□Not difficult □Mildly difficult □Moderately difficult □Considerably pain □Extremely pain
6. To what extent has it been difficult to stand up from a chair?
□Not difficult □Mildly difficult □Moderately difficult □Considerably pain □Extremely pain
7. To what extent has it been difficult to walk inside the house?
□Not difficult □Mildly difficult □Moderately difficult □Considerably pain □Extremely pain
8. To what extent has it been difficult to put on and take off shirts?
□Not difficult □Mildly difficult □Moderately difficult □Considerably pain □Extremely pain
9. To what extent has it been difficult to put on and take off trousers and pants?
□Not difficult □Mildly difficult □Moderately difficult □Considerably pain □Extremely pain
10. To what extent has it been difficult to use the toilet?
□Not difficult □Mildly difficult □Moderately difficult □Considerably pain □Extremely pain
11. To what extent has it been difficult to wash your body in the bath?
□Not difficult □Mildly difficult □Moderately difficult □Considerably pain □Extremely pain
12. To what extent has it been difficult to go up and down stairs?
□Not difficult □Mildly difficult □Moderately difficult □Considerably pain □Extremely pain
13. To what extent has it been difficult to walk briskly?
□Not difficult □Mildly difficult □Moderately difficult □Considerably pain □Extremely pain
14. To what extent has it been difficult to keep yourself?
□Not difficult □Mildly difficult □Moderately difficult □Considerably pain □Extremely pain
15. How far can you keep walking without rest? (please select the closest answer)
□Not difficult □Mildly difficult □Moderately difficult □Considerably pain □Extremely pain
16. To what extent has it been difficult to go out to visit neighbors?
□Not difficult □Mildly difficult □Moderately difficult □Considerably pain □Extremely pain
17. To what extent has it been difficult to carry objects weighing approximately 2 kg (2 standard milk bottles or 2 PET bottles each containing 1 L)?
□Not difficult □Mildly difficult □Moderately difficult □Considerably pain □Extremely pain
18. To what extent has it been difficult to go out using public transportation?
□Not difficult □Mildly difficult □Moderately difficult □Considerably pain □Extremely pain
19. To what extent have simple tasks and homework (preparing meals, cleaning up, etc.) been difficult?
□Not difficult □Mildly difficult □Moderately difficult □Considerably pain □Extremely pain
20. To what extent have load‐bearing tasks and housework (cleaning the yard, carrying heavy bedding, etc.) been difficult?
□Not difficult □Mildly difficult □Moderately difficult □Considerably pain □Extremely pain
21. To what extent has it been difficult to perform sports activity (jogging, swimming, gate ball, dancing, etc.)?
□Not difficult □Mildly difficult □Moderately difficult □Considerably pain □Extremely pain
22. Have you been restricted from meeting your friends?
□Not restricted □Slightly restricted □Restricted about half the time □Considerably restricted □Give up all activity
23. Have you been restricted from joining social activities (meeting friends, playing sport, engaging in activities and hobbies, etc.)?
□Not restricted □Slightly restricted □Restricted about half the time □Considerably restricted □Give up all activity
24. Have you ever felt anxious falls in your house?
□Have not felt anxious □Have occasionally felt anxious □Have sometimes felt anxious □Have often felt anxious □Have constantly felt anxious
25. Have you ever felt anxious about being unable to walk in the future?
□Have not felt anxious □Have occasionally felt anxious □Have sometimes felt anxious □Have often felt anxious □Have constantly felt anxious

### 2.4 Statistical Analysis

Continuous variables (e.g., age, BMI, GLFS‐25 score) were assessed for normality using the Shapiro–Wilk test. When the data followed a normal distribution, a two‐sample *t*‐test was used; otherwise, the Mann–Whitney U test was applied. Categorical variables (e.g., sex, locomotive syndrome stage) were compared using the chi‐square test. Statistical significance was set at *p* < 0.05. All analyses were performed using JMP Pro 17 (SAS Institute Inc., Cary, NC, USA). Prior to the IRT analysis, principal component analysis was performed separately for the older adult group and the younger and middle‐aged adult group to examine the unidimensionality of the GLFS‐25.

In accordance with the IRT, a higher score was operationally defined as indicating more severe LS (i.e., the latent trait value must be high to be selected). The graded response model (GRM) was applied because it is theoretically suitable for ordered Likert‐type items representing cumulative severity. Compared with alternative polytomous models such as the generalized partial credit model, the GRM models cumulative probabilities across ordered thresholds and provides stable parameter estimation even when upper categories are rarely endorsed. Parameter estimation was performed using the maximum likelihood method based on the expectation–maximization algorithm. Category characteristic curves (CCCs) were drawn for each item, and discrimination and difficulty were calculated. Representative items presented in the main text were defined as those with moderate‐to‐high discrimination (a ≥ 1.0) and clear visual separation between response categories. Items with a 0% response rate in one or more response categories were retained in the analysis to ensure consistency across age groups. Although these categories did not contribute to parameter estimation, they did not prevent model convergence and were interpreted with caution. According to Baker's classification, discrimination parameters (a) between 0.35 and 1.69 indicate low to high discrimination, while values ≥ 1.70 represent very high discrimination. In practice, values between 0.5 and 2.5 are often considered acceptable as an empirical range in applied IRT studies. Difficulty parameters (b) typically range from −3 to +3, corresponding to the effective range of the latent trait (θ) under a standard normal assumption [19]. The IRT analysis software Exametrika ver 5.6 (Shojima, Tokyo, Japan) was used. Model–data fit was evaluated using the root mean square error of approximation (RMSEA), which is widely applied in IRT research to assess overall and item‐level fit. RMSEA values below 0.06 indicate good fit, and values below 0.08 are considered acceptable [20]. Within each group, the latent trait (θ) was standardized with a mean of 0 and a standard deviation of 1. Exametrika automatically applies this scaling under Samejima's graded response model to ensure model identification and stable estimation. Therefore, no linking or equating procedure across groups was required. To statistically evaluate the differences in item parameters between the two age groups, Wald tests were conducted to compare discrimination (a) and difficulty threshold (b) parameters for each item, based on the standard errors estimated from the IRT model. Statistical significance was set at *p* < 0.05. Furthermore, to assess the robustness of the 65‐year cutoff, sensitivity analyses were performed using alternative age thresholds of 60, 70, and 75 years. For each threshold, separate IRT models were estimated for the younger and middle‐aged adults and the older adults, and the same Wald test procedure was applied to compare discrimination parameters across age thresholds. These analyses were performed using JMP Pro 17.

## 3 Results

**FIGURE 1:**
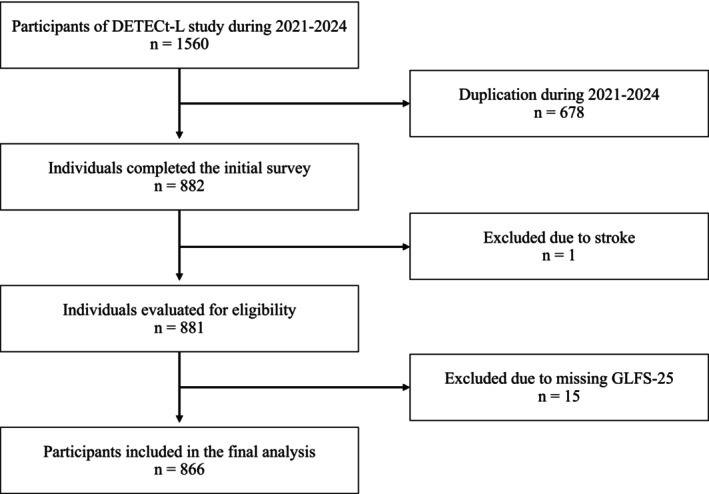
Participant flow diagram of 1560 participants; 866 were included in the final analysis after applying the exclusion criteria. DETECt‐L study, study for the diagnosis, early detection, and treatment of locomotive syndrome using an epidemiological cohort study; LS, locomotive syndrome.

A total of 1560 individuals participated in the DETECt‐L study during 2021–2024. After excluding 678 duplicated entries, 882 individuals completed the initial survey. One individual was excluded due to a history of stroke, and 15 were excluded due to missing responses to the GLFS‐25. Consequently, 866 participants (183 men, 683 women) were included in the final analysis (Figure 1). The younger and middle‐aged adults group consisted of 322 (men: 79, women: 243) participants, and the older adults group consisted of 544 (men: 104, women: 440), with a GLFS‐25 score of 6.4 ± 6.2 points for the younger and middle‐aged adult group and 8.5 ± 8.6 points for the older adults group (Table 2).

**TABLE 2:** Participant characteristics by age group.

	All (*n* = 866)	Younger and middle‐aged adults (*n* = 322)	Older adults (*n* = 544)	*p*
Sex	Men: 183, Women: 683	Men: 79, Women: 243	Men: 104, Women: 440	0.06
Age	64.6 (14.9)	48.2 (11.2)	74.3 (5.2)	0.0001
BMI (kg/m^2^)	22.6 (3.1)	22.3 (3.4)	22.7 (2.9)	0.02
GLFS‐25	7.7 (7.9)	6.4 (6.2)	8.5 (8.6)	0.002
LS classification (%)	non: 484, 1: 283, 2: 56, 3: 43 (non: 56, 1: 33, 2: 6, 3: 5)	non: 200, 1: 104, 2: 12, 3: 6 (non: 62, 1: 32, 2: 4, 3: 2)	non:284, 1: 179, 2: 44, 3: 37 (non: 52, 1: 33, 2: 8, 3: 7)	0.0003

*Note:* Values are presented as mean (standard deviation). All continuous variables were non‐normally distributed, and the Mann–Whitney U test was applied. Categorical variables were compared using the chi‐square test. A *p*‐value < 0.05 was considered statistically significant.

Abbreviations: BMI, body mass index; GLFS‐25, 25‐question geriatric locomotive function scale; LS, locomotive syndrome, LS classification was based on GLFS‐25 total score.

Principal component analysis confirmed the unidimensional structure of the GLFS‐25 in both age groups.

In the older adult group, the first principal component had an eigenvalue of 9.47, accounting for 37.88% of the total variance. In the younger and middle‐aged group, the first principal component had an eigenvalue of 8.24, accounting for 32.97% of the variance. In both groups, scree plots showed a clear drop after the first component, supporting a single‐factor structure. Based on this assumption, IRT analysis revealed that in the younger and middle‐aged adult group, all items except Item 8 demonstrated acceptable discrimination (*a* ≥ 0.5). Among these, Items 3 (lower limb pain, *a* = 1.00, *b*_1_ = −0.80), 4 (pain, *a* = 1.16, *b*_1_ = −0.68), 21 (sports, *a* = 1.03, *b*_1_ = −0.41), and 23 (social activities, *a* = 1.07, *b*_1_ = −0.51) exhibited moderate‐to‐high discrimination (*a* ≥ 1.0). These items also showed relatively low difficulty values (*b*_1_ < −0.5), suggesting their potential utility in detecting early signs of LS in this age group. All items showed acceptable difficulty parameters (*b* values between −4 and + 4). However, response rates for category “3” were 0% in Items 5–8, 10, 11, 16, 19, and 20, and for category “4” in Items 3–7, 9, 10–21, and 24.

In the older adults group, all items except Item 22 demonstrated acceptable discrimination. Items with moderate‐to‐high discrimination included Item 1 (upper limb pain, *a* = 1.04, *b*_1_ = −0.75), 3 (lower limb pain, *a* = 1.11, *b*_1_ = −0.63), 4 (pain, *a* = 1.19, *b*_1_ = −0.60), 12 (stairs, *a* = 1.07, *b*_1_ = −0.38), 13 (brisk walking, *a* = 1.11, *b*_1_ = −0.43), 20 (housework, *a* = 1.08, *b*_1_ = −0.41), 21 (sports, *a* = 1.14, *b*_1_ = −0.32), and 25 (anxiety about walking, *a* = 1.26, *b*_1_ = −0.49). These items showed moderate‐to‐high discrimination and moderate difficulty, indicating their relevance for screening LS among older adults. All items had acceptable difficulty values. Items 8, 10, 14, and 19 had 0% response rates for category “3,” and Items 1, 3, 7–11, 14, 19, 20, and 24 had 0% response rates for category “4” (Table 3).

**TABLE 3:** Item response theory results for younger and middle‐aged adults and older adults.

Younger and middle‐aged adults											Older adults									
	Percentage of GLFS‐25 responses					Discrimination (*a*)	Difficulty				Percentage of GLFS‐25 responses					Discrimination (*a*)	Difficulty			
	0	1	2	3	4		*b*_1_	*b*_2_	*b*_3_	*b*_4_	0	1	2	3	4		*b*_1_	*b*_2_	*b*_3_	*b*_4_
Item1	51.6	37.6	7.8	1.9	1.2	0.97	−0.66	1.27	2.15	2.60	47.6	39.2	9.4	3.9	0	1.04	−0.66	1.35	2.39	—
Item2	50.6	38.2	9.9	0.9	0.3	0.80	−0.73	1.31	2.63	3.07	51.5	35.1	10.3	2.8	0.4	0.80	−0.54	1.23	2.30	3.16
Item3	59.9	31.7	6.5	1.9	0	1.00	−0.31	1.61	2.64	—	46.7	38.4	11.2	3.7	0	1.11	−0.67	1.19	2.43	—
Item4	61.8	33.9	3.7	0.6	0	1.16	−0.30	1.83	3.01	—	60.1	29.6	8.1	2.0	0.2	1.19	−0.21	1.24	2.25	3.36
Item5	92.2	7.5	0.3	0	0	0.72	1.45	3.22	—	—	80.7	15.8	2.4	0.9	0.2	0.85	0.67	1.94	2.45	3.10
Item6	95.7	3.7	0.6	0	0	0.63	1.84	2.83	—	—	81.6	14.5	3.1	0.4	0.4	0.75	0.73	1.88	2.56	2.79
Item7	98.1	1.6	0.3	0	0	0.72	2.23	2.98	—	—	89.9	9.0	0.7	0.4	0	0.79	1.22	2.44	2.85	—
Item8	95.7	3.4	0.6	0	0.3	0.49	1.89	2.50	—	2.79	90.6	8.8	0.6	0	0	0.64	1.58	3.10	—	—
Item9	94.4	4.3	0.9	0.3	0	0.86	1.48	2.35	2.98	—	80.0	18.0	1.8	0.2	0	0.92	0.67	2.33	3.27	—
Item10	97.5	1.9	0.6	0	0	0.59	2.14	2.72	—	—	92.6	7.0	0.4	0	0	0.63	1.72	3.19	—	—
Item11	96.6	2.2	1.2	0	0	0.75	1.93	2.41	—	—	92.5	6.8	0.6	0.2	0	0.66	1.57	2.65	3.05	—
Item12	86.3	12.1	1.2	0.3	0	0.72	0.93	2.37	2.99	—	63.2	28.9	5.1	2.6	0.2	1.07	−0.10	1.44	2.12	3.38
Item13	86.6	11.2	1.6	0.6	0	0.77	0.90	2.16	2.72	—	62.1	27.8	7.0	2.9	0.2	1.11	−0.13	1.27	2.06	3.32
Item14	96.0	3.1	0.6	0.3	0	0.68	1.82	2.50	2.89	—	92.8	6.8	0.4	0	0	0.71	1.68	3.16	—	—
Item15	69.8	28.5	1.1	0.7	0	0.75	0.08	2.36	2.71	—	61.8	29.0	4.6	3.3	1.3	0.83	−0.13	1.46	1.94	2.58
Item16	97.5	1.6	0.9	0	0	0.57	2.13	2.53	—	—	91.7	7.0	0.4	0.7	0.2	0.68	1.39	2.30	2.42	2.93
Item17	91.0	7.8	0.9	0.3	0	0.65	1.32	2.47	2.97	—	74.8	20.8	2.6	1.5	0.4	0.99	0.40	1.83	2.27	2.88
Item18	96.3	2.8	0.3	0.6	0	0.59	1.84	2.42	2.55	—	87.3	10.3	1.5	0.7	0.2	0.85	1.04	2.10	2.51	3.09
Item19	96.3	3.1	0.6	0	0	0.60	2.00	2.80	—	—	90.8	7.4	1.8	0	0	0.79	1.37	2.37	—	—
Item20	88.8	10.6	0.6	0	0	0.76	1.16	3.01	—	—	72.4	23.7	2.4	1.5	0	1.08	0.27	1.88	2.38	—
Item21	67.4	26.7	4.7	1.2	0	1.03	−0.03	1.74	2.72	—	60.7	32.0	4.8	2.0	0.6	1.14	−0.18	1.50	2.14	2.84
Item22	59.3	20.5	6.8	7.5	5.9	0.95	−0.24	0.81	1.28	2.11	73.7	21.5	2.2	2.2	0.4	0.43	0.36	1.89	2.16	2.68
Item23	51.6	24.5	6.2	8.4	9.3	1.07	−0.51	0.67	1.07	1.91	68.9	23.7	3.7	2.2	1.5	0.54	0.19	1.59	1.95	2.30
Item24	91.0	8.4	0.3	0.3	0	0.74	1.26	2.62	2.84	—	69.3	29.0	1.1	0.6	0	0.86	0.26	2.38	2.75	—
Item25	61.5	33.2	3.7	1.2	0.3	0.79	−0.31	1.76	2.44	3.07	46.9	47.1	2.8	2.8	0.6	1.26	−0.59	1.67	2.04	2.87

*Note:* Percentages of responses for each GLFS‐25 item are shown by category (0–4). Discrimination parameters (*a*) and item difficulty parameters (*b*_1_–*b*_4_) are presented for each item. “—” indicates that the parameter could not be estimated due to 0% response.

**FIGURE 2:**
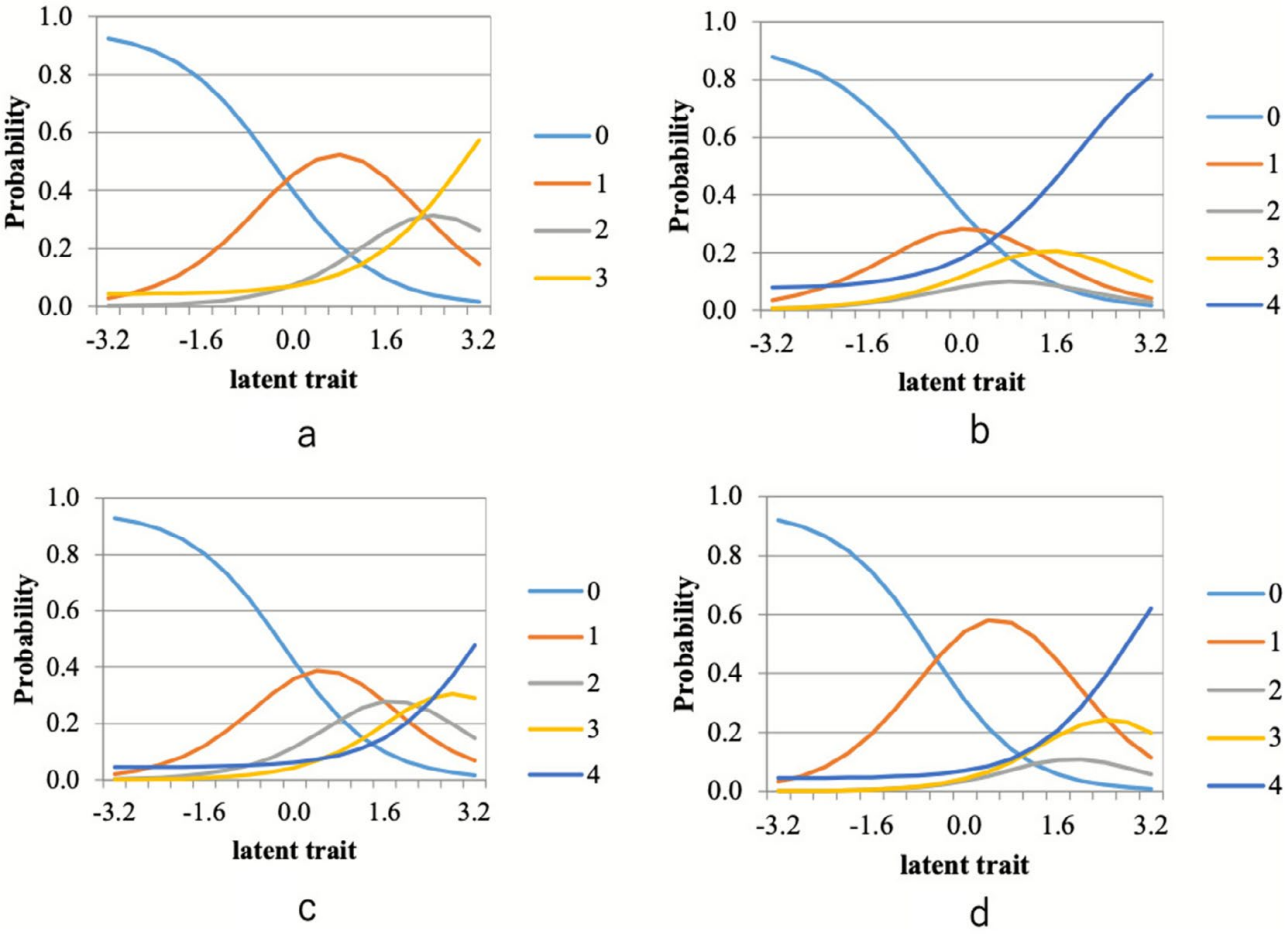
(a) Category characteristic curve for Item 4 in younger and middle‐aged adults. (b) Category characteristic curve for Item 23 in younger and middle‐aged adults. (c) Category characteristic curve for Item 4 in older adults. (d) Category characteristic curve for Item 25 in older adults.

To visually represent item functioning across groups, CCCs were generated for key items with moderate‐to‐high discrimination. In younger and middle‐aged adults, Items 4 and 23 demonstrated steep and distinct response transitions across theta levels, while in older adults, Items 4 and 25 showed similarly defined curves. These visual patterns were consistent with the item‐level IRT estimates, suggesting that the discriminative contribution of individual items may differ between age groups (Figure 2). Representative CCCs for these items are presented in Figure 2, illustrating the differences in response probability across latent trait levels. For comprehensive visualization, CCCs for all 25 items are provided in the Supporting Information, presented separately for younger and middle‐aged (Figure S1) and older adults (Figure S2).

The model–data fit indices indicated acceptable fit for both groups (RMSEA = 0.04 for younger and middle‐aged adults, RMSEA = 0.05 for older adults). At the item level, Item 13 showed the highest RMSEA (0.08) in younger and middle‐aged adults, while Items 17 (0.08) and 23 (0.09) had the highest values in older adults. Detailed item‐level RMSEA values are provided in the Supporting Information (Table S1).

Following the IRT analyses, Wald tests were conducted to statistically compare item parameters between younger (< 65 years) and older (≥ 65 years) adults. The results are summarized in Table 4, showing *Z*‐values and *p*‐values for discrimination (*a*) and threshold (*b*_1_–*b*_4_) parameters. Significant differences (*p* < 0.05) were observed for Items 12, 13, 17, 18, 20, and 25 (higher in older adults) and Items 22 and 23 (higher in younger and middle‐aged adults). These findings provide formal statistical evidence supporting the presence of distinct item‐response patterns between age groups. Although formal anchor‐based Differential Item Functioning testing was not separately performed, the Wald test results provide comparable evidence of differential item functioning between age groups.

**TABLE 4:** Wald test results for item parameter differences between younger (< 65 years) and older (≥ 65 years) adults.

	*Z* (*a*)	*p* (*a*)	*Z* (*b*_1_)	*p* (*b*_1_)	*Z* (*b*_2_)	*p* (*b*_2_)	*Z* (*b*_3_)	*p* (*b*_3_)	*Z* (*b*_4_)	*p* (*b*_4_)
Item1	0.56	0.57	0.01	0.99	0.34	0.73	0.73	0.46	0.19	0.85
Item2	0.98	0.33	1.03	0.30	−0.33	0.74	−1.04	0.30	N/A	N/A
Item3	0.77	0.44	−2.23	**< 0.05**	−1.52	0.13	−0.45	0.65	N/A	N/A
Item4	0.23	0.81	0.59	0.56	−1.98	**< 0.05**	−1.26	0.21	N/A	N/A
Item5	1.00	0.32	−2.43	**< 0.05**	−2.19	**< 0.05**	N/A	N/A	N/A	N/A
Item6	0.86	0.39	−3.47	**< 0.05**	−2.20	**< 0.05**	N/A	N/A	N/A	N/A
Item7	0.46	0.64	−2.97	**< 0.05**	−1.25	0.21	N/A	N/A	N/A	N/A
Item8	1.33	0.18	−1.04	0.30	1.51	0.13	N/A	N/A	N/A	N/A
Item9	0.43	0.67	−3.22	**< 0.05**	−0.05	0.96	0.58	0.56	N/A	N/A
Item10	0.28	0.78	−1.22	0.22	0.97	0.33	N/A	N/A	N/A	N/A
Item11	−0.63	0.53	−1.21	0.23	0.73	0.47	N/A	N/A	N/A	N/A
Item12	2.75	**< 0.05**	−4.53	**< 0.05**	−3.15	**< 0.05**	−2.24	**< 0.05**	N/A	N/A
Item13	2.55	**< 0.05**	−4.79	**< 0.05**	−3.28	**< 0.05**	−1.86	0.06	N/A	N/A
Item14	0.18	0.86	−0.47	0.64	1.55	0.12	N/A	N/A	N/A	N/A
Item15	0.65	0.52	−1.16	0.25	−2.52	**< 0.05**	−2.53	**< 0.05**	N/A	N/A
Item16	0.85	0.40	−2.87	**< 0.05**	−0.83	0.41	N/A	N/A	N/A	N/A
Item17	2.65	**< 0.05**	−3.79	**< 0.05**	−2.31	**< 0.05**	−2.06	**< 0.05**	N/A	N/A
Item18	2.06	**< 0.05**	−3.70	**< 0.05**	−1.65	0.10	−0.22	0.83	N/A	N/A
Item19	1.36	0.17	−1.91	0.06	−0.97	0.33	N/A	N/A	N/A	N/A
Item20	2.22	**< 0.05**	−3.08	**< 0.05**	−1.99	**< 0.05**	N/A	N/A	N/A	N/A
Item21	0.78	0.44	−0.88	0.38	−0.85	0.40	−1.26	0.21	N/A	N/A
Item22	−4.65	**< 0.05**	2.82	**< 0.05**	6.38	**< 0.05**	4.72	**< 0.05**	1.77	0.08
Item23	−4.39	**< 0.05**	3.94	**< 0.05**	5.96	**< 0.05**	5.25	**< 0.05**	1.32	0.19
Item24	0.96	0.34	−4.23	**< 0.05**	−1.07	0.28	−0.34	0.73	N/A	N/A
Item25	3.88	**< 0.05**	−1.56	0.12	−0.40	0.69	−1.55	0.12	−0.47	0.64

*Note:* Wald tests were conducted to compare item parameters between younger (< 65 years) and older (≥ 65 years) adults. *p* < 0.05 considered statistically significant (bolded). *Z* (*a*) represents discrimination parameters, and *Z* (*b*_1_–*b*_4_) represent threshold parameters. Positive *Z*‐values indicate higher parameter estimates in older adults, whereas negative *Z*‐values indicate higher estimates in younger and middle‐aged adults.

Abbreviations: N/A, indicates that the corresponding threshold parameter was not estimated due to limited category responses; *Z*, Wald statistic.

In addition, sensitivity analyses were conducted using alternative age thresholds of 60, 70, and 75 years to assess the robustness of the 65‐year cutoff. The results are summarized in Table 5. Similar patterns of item discrimination were observed for the 60‐ and 65‐year thresholds, indicating stable findings across these cutoffs. At the 70‐year cutoff, a greater number of items showed significant between‐group differences, whereas at 75 years, the number of significant items decreased slightly.

**TABLE 5:** Sensitivity analysis of discrimination parameters (*a*) using alternative age thresholds (60, 65, 70, and 75 years).

	60 years		65 years		70 years		75 years	
	*Z* (*a*)	*p* (*a*)	*Z* (*a*)	*p* (*a*)	*Z* (*a*)	*p* (*a*)	*Z* (*a*)	*p* (*a*)
Item1	−0.44	0.66	0.56	0.57	0.49	0.63	2.25	**< 0.05**
Item3	1.82	0.07	0.77	0.44	2.03	**< 0.05**	−0.03	0.98
Item5	1.55	0.12	1.00	0.32	2.39	**< 0.05**	1.89	0.06
Item6	0.76	0.45	0.86	0.39	2.46	**< 0.05**	1.32	0.19
Item7	1.23	0.22	0.46	0.64	2.47	**< 0.05**	2.08	**< 0.05**
Item8	1.60	0.11	1.33	0.18	2.41	**< 0.05**	2.06	**< 0.05**
Item12	3.29	**< 0.05**	2.75	**< 0.05**	3.08	**< 0.05**	2.59	**< 0.05**
Item13	2.84	**< 0.05**	2.55	**< 0.05**	2.94	**< 0.05**	2.25	**< 0.05**
Item15	0.18	0.86	0.65	0.52	−0.03	0.98	−2.03	**< 0.05**
Item17	3.15	**< 0.05**	2.65	**< 0.05**	2.01	**< 0.05**	1.83	0.07
Item18	2.68	**< 0.05**	2.06	**< 0.05**	3.01	**< 0.05**	2.14	**< 0.05**
Item19	1.55	0.12	1.36	0.17	2.69	**< 0.05**	2.01	**< 0.05**
Item20	2.20	**< 0.05**	2.22	**< 0.05**	3.30	**< 0.05**	3.04	**< 0.05**
Item22	−4.22	**< 0.05**	−4.65	**< 0.05**	−3.16	**< 0.05**	−1.02	0.31
Item23	−3.81	**< 0.05**	−4.39	**< 0.05**	−1.44	0.15	−0.60	0.55
Item25	4.35	**< 0.05**	3.88	**< 0.05**	1.89	0.06	1.51	0.13

*Note:* *p* < 0.05 is considered statistically significant (bolded). Items that showed statistically significant differences (*p* < 0.05) in discrimination parameters between younger and older adults at any cutoff are presented. Positive *Z*‐values indicate higher discrimination in older adults, and negative *Z*‐values indicate higher discrimination in younger and middle‐aged adults. Statistical significance was determined using Wald tests based on standard errors estimated from the IRT model.

Abbreviation: *Z*, Wald statistic.

## 4 Discussion

Most of the GLFS‐25 items demonstrated acceptable discrimination and difficulty values using IRT. Item‐level patterns, however, differed by age group: pain‐related items (e.g., lower limb pain, pain during movement) were more discriminative among younger and middle‐aged adults, whereas activities of daily living and anxiety items (e.g., difficulty with stairs, anxiety about walking) were more discriminative among older adults. Furthermore, the results of the Wald tests statistically confirmed these item‐level differences, supporting the presence of distinct response patterns between age groups observed in the IRT analyses. Sensitivity analyses using alternative age thresholds (60, 70, and 75 years) showed similar discrimination trends across cutoffs, confirming the robustness of the 65‐year division. These results indicate that the 65‐year cutoff appropriately captures a meaningful transition in locomotive function between middle‐aged and older adults. These findings indicate that different aspects of locomotive function decline are more salient at different life stages and support age‐adapted screening strategies.

The novelty of this study lies in its being the first to directly compare GLFS‐25 item characteristics between younger (< 65 years) and older (≥ 65 years) adults by combining IRT with formal statistical testing. This dual approach enabled us to distinguish items that were highly discriminative within each age group from those that differed between age groups. In the IRT analyses, pain‐related and social participation items (e.g., Items 3, 4, 21, and 23) showed moderate‐to‐high discrimination among younger adults, whereas mobility‐ and anxiety‐related items (e.g., Items 12, 13, 20, and 25) were more discriminative among older adults. The Wald tests further demonstrated that several of these items—particularly Items 12, 13, 17, 18, 20, and 25—had significantly higher parameter estimates in older adults, while Items 22 and 23 showed higher estimates in younger adults. This combination of sensitivity (from IRT) and statistical validation (from Wald tests) provides a robust, data‐driven foundation for developing age‐tailored short forms and computer‐adaptive testing for locomotive syndrome.

Although the GLFS‐25 is effective for detecting severe LS [9, 10], its ability to capture early‐stage LS remains limited. In younger and middle‐aged adults, pain (Items 3 and 4) and social participation (Items 21 and 23) items had moderate‐to‐high discrimination, while Item 8 (difficulty putting on shirts) showed a ceiling effect, likely due to preserved function and cultural clothing factors. In older adults, pain, mobility, and ADL items (e.g., Items 1, 4, 12, and 25) had moderate‐to‐high discrimination, whereas Item 22 (restricted from meeting friends) was less informative, possibly reflecting social restrictions unrelated to physical function. From a clinical standpoint, items that showed both moderate‐to‐high discrimination and statistically significant differences in the Wald tests—particularly Items 12, 13, 17, 18, 20, and 25 in older adults and Items 22 and 23 in younger and middle‐aged adults—represent key indicators of functional transition across age groups. The appearance of more significant item‐level differences beyond 70 years further suggests that locomotive function becomes increasingly heterogeneous in late older adulthood. Overall, younger adults were more sensitive to pain‐related decline, whereas older adults were more responsive to broader functional and anxiety‐related limitations. Because the GLFS‐25 was originally validated for geriatric populations, its use in younger cohorts requires caution; however, items with moderate‐to‐high discrimination and low difficulty thresholds (e.g., *b*_1_ < −0.5) may help detect early‐stage LS. Previous simplified GLFS‐based tools [21, 22] did not include pain items, which our findings suggest are essential in younger populations. Thus, an age‐sensitive short form of 5–8 items, combining pain/social participation items for younger adults and ADL‐anxiety items for older adults, could be clinically feasible as a brief 3‐min screening tool. Item parameters may also serve as priors for computer‐adaptive testing.

Response patterns revealed ceiling effects, with categories “3” and “4” rarely chosen, especially in younger adults. Zero‐response categories challenged parameter estimation and reduced the stability of IRT estimates, though model convergence was not prevented. Sparse responses in younger adults likely reflected the low prevalence of severe limitations. Future studies could consider collapsing categories or applying alternative IRT models.

Items with moderate‐to‐high discrimination and low difficulty thresholds were most useful for early LS detection. A few items consistently showed strong discrimination, whereas Item 8 in younger adults and Item 22 in older adults were less suitable. These results support developing a shorter, age‐specific GLFS‐25.

This study has four limitations. First, sampling bias: LS prevalence in older adults was higher in our study (48%) than in previous reports (36.4%) [23], while prevalence of stage 2–3 LS in younger adults was lower (6% vs. 12.4%) [24]. This may explain sparse responses in younger adults. Second, reliability concerns: prior reliability studies of the GLFS‐25 involved only older adults [7], therefore, its validity in younger groups is uncertain. Third, sample representativeness: the younger group mainly included adults in their 40s–60s, with few in their 20s–30s, limiting generalizability. Fourth, although several items showed sparse upper‐category responses (“3” and “4”), particularly among younger adults, we did not perform a category‐collapse sensitivity analysis because models with different category structures are not directly comparable in the same IRT framework. However, the estimation process in the IRT algorithm automatically treats such items as having fewer effective response levels, ensuring stable parameter estimation. Although a few items exhibited slightly higher RMSEA values (0.08–0.09), the overall model fit was satisfactory; therefore, these minor deviations were considered negligible and did not indicate systematic misfit.

In conclusion, IRT analysis revealed distinct patterns of item discrimination across age groups. Pain‐related items were most informative in younger adults, whereas ADL and anxiety items were more relevant in older adults. These findings support the GLFS‐25's psychometric validity across adulthood and provide a rationale for age‐adapted screening tools, including short forms and adaptive formats. A potential short‐form could prioritize pain/social participation items (younger) and mobility/anxiety items (older), serving as a rapid 3–5‐min screening instrument in primary care or community health. Further studies should validate these tools in diverse settings.

## Author Contributions

S.Y. contributed to the study design, data collection and analysis, and drafted the manuscript. K.H. contributed to data acquisition and provided critical revisions of the manuscript. J.N., K.H., and R.T. provided conceptual advice and assisted in manuscript revisions. All authors have read and approved the final version of the manuscript.

## Funding

This work was supported by the Ministry of Health, Labour and Welfare (MHLW) FA Program under Grant Number (JP22FA1003).

## Ethics Statement

This study was conducted in accordance with the principles of the Declaration of Helsinki. The research protocol was approved by the Epidemiological Research Ethics Committee of Hiroshima University (approval number: E2022‐0086). All parameters measured in this study were noninvasive and essential for assessing the participants' functional status.

## Consent

Written informed consent was obtained from all participants.

## Conflicts of Interest

The authors declare no conflicts of interest.

## Supporting information


**Figure S1:** Category characteristic curves for all 25 GLFS‐25 items, presented separately for younger and middle‐aged adults.

**Figure S2:** Category characteristic curves for all 25 GLFS‐25 items, presented separately for older adults.

**Table S1:** Item‐level model fit indices for younger and middle‐aged and older adults.

## Data Availability

The data that support the findings of this study are available on request from the corresponding author. The data are not publicly available due to privacy or ethical restrictions.

## References

[1] H. Ishibashi , “Locomotive Syndrome in Japan,” Osteoporosis and Sarcopenia 4 (2018): 86–94, 10.1016/j.afos.2018.09.004. 30775549 PMC6362958

[2] Cabinet Office, Government of Japan. Headquarters for Measures for an Aging Society , Annual Report on the Aging Society (Cabinet Office, Government of Japan, 2023), https://www8.cao.go.jp/kourei/english/annualreport/2023/pdf/2023.pdf.

[3] K. Kobayashi , S. Imagama , K. Ando , et al., “Locomotive Syndrome Stage 1 Predicts Significant Worsening of Future Motor Performance: The Prospective Yakumo Study,” BioMed Research International 2019 (2019): 1970645, 10.1155/2019/1970645. 31687379 PMC6794969

[4] N. Yoshimura , S. Muraki , H. Oka , et al., “Association Between New Indices in the Locomotive Syndrome Risk Test and Decline in Mobility: Third Survey of the ROAD Study,” Journal of Orthopaedic Science 20 (2015): 896–905, 10.1007/s00776-015-0741-5. 26104219 PMC4575347

[5] N. Yoshimura , T. Iidaka , C. Horii , et al., “Epidemiology of Locomotive Syndrome Using Updated Clinical Decision Limits: 6‐Year Follow‐Ups of the ROAD Study,” Journal of Bone and Mineral Metabolism 40 (2022): 623–635, 10.1007/s00774-022-01324-8. 35536512

[6] Y. Sawaya , T. Hirose , S. Onuma , et al., “Prevalence and Associated Factors of Locomotive Syndrome in Young Japanese Adults: A Cross‐Sectional Study,” BMC Musculoskeletal Disorders 25 (2024): 366, 10.1186/s12891-024-07493-z. 38730399 PMC11084025

[7] A. Seichi , Y. Hoshino , T. Doi , M. Akai , Y. Tobimatsu , and T. Iwaya , “Development of a Screening Tool for Risk of Locomotive Syndrome in the Elderly: The 25‐Question Geriatric Locomotive Function Scale,” Journal of Orthopaedic Science 17 (2012): 163–172, 10.1007/s00776-011-0193-5. 22222445

[8] C. Wang , T. Ikemoto , A. Hirasawa , Y. C. Arai , S. Kikuchi , and M. Deie , “Assessment of Locomotive Syndrome Among Older Individuals: A Confirmatory Factor Analysis of the 25‐Question Geriatric Locomotive Function Scale,” PeerJ 8 (2020): e9026, 10.7717/peerj.9026. 32328357 PMC7164427

[9] T. Kobayashi , T. Morimoto , C. Shimanoe , R. Ono , K. Otani , and M. Mawatari , “Clinical Characteristics of Locomotive Syndrome Categorised by the 25‐Question Geriatric Locomotive Function Scale: A Systematic Review,” BMJ Open 13 (2023): e068645, 10.1136/bmjopen-2022-068645. PMC10193047 37192799

[10] S. Inanaga , M. Hasegawa , M. Kosuge , S. Ichimura , T. Morii , and N. Hosogane , “Relationship Between the 25‐Question Geriatric Locomotive Function Scale and Physical Function in the Elderly People,” Journal of Bone and Mineral Metabolism 41 (2023): 550–556, 10.1007/s00774-023-01427-w. 37029834

[11] A. Suzuki , H. Toyoda , and S. Kosugi , “Construction of a Stress Reaction Scale Based on Item Response Model and Examination of the Developmental Process of Psychological Stress Reactions by Using Test Characteristic Curves (In Japanese),” Shinrigaku Kenkyu 75 (2004): 389–396, 10.4992/jjpsy.75.389. 15747561

[12] L. B. Mokkink , C. B. Terwee , D. L. Patrick , et al., “The COSMIN Checklist for Assessing the Methodological Quality of Studies on Measurement Properties of Health Status Measurement Instruments: An International Delphi Study,” Quality of Life Research 19 (2010): 539–549, 10.1007/s11136-010-9606-8. 20169472 PMC2852520

[13] L. B. Mokkink , H. C. W. de Vet , C. A. C. Prinsen , et al., “COSMIN Risk of Bias Checklist for Systematic Reviews of Patient‐Reported Outcome Measures,” Quality of Life Research 27 (2018): 1171–1179, 10.1007/s11136-017-1765-4. 29260445 PMC5891552

[14] S. Sakate , “Actual Status of Locomotive Syndrome Among Elderly Females With Regular Exercise Habits (In Japanese),” JAMHTS 47 (2020): 345–351, 10.7143/jhep.47.345.

[15] T. Iwaya , M. Akai , and T. Doi , “Operationalistic Approach for Locomotive Syndrome−we Don't Know What Locomo Really Is (In Japanese),” Bone and Joint Nerve 4 (2014): 393–401.

[16] T. Kato , A. Nishimura , M. Ohtsuki , et al., “Is Musculoskeletal Pain Related to Locomotive Syndrome Even in Young and Middle‐Aged Adults?,” Modern Rheumatology 32 (2022): 213–220, 10.1080/14397595.2021.1906512. 33769924

[17] Y. Ishihara , H. Ozaki , T. Nakagata , et al., “Association Between Daily Physical Activity and Locomotive Syndrome in Community‐Dwelling Japanese Older Adults: A Cross‐Sectional Study,” International Journal of Environmental Research and Public Health 19 (2022): 8164, 10.3390/ijerph19138164. 35805823 PMC9265950

[18] K. A. Nakamura , “‘Super‐Aged’ Society and the “Locomotive Syndrome”,” Journal of Orthopaedic Science 13 (2008): 1–2, 10.1007/s00776-007-1202-6. 18274847 PMC2779431

[19] F. B. Baker , The Basics of Item Response Theory, 2nd ed. (Educational Resources Information Center Clearinghouse on Assessment and Evaluation, 2001), https://eric.ed.gov/?id=ED458219.

[20] L. T. Hu and P. M. Bentler , “Cutoff Criteria for Fit Indexes in Covariance Structure Analysis: Conventional Criteria Versus New Alternatives,” Structural Equation Modeling: A Multidisciplinary Journal 6 (1999): 1–55, 10.1080/10705519909540118.

[21] T. Kobayashi , T. Morimoto , C. Shimanoe , R. Ono , K. Otani , and M. Mawatari , “Development of a Simple Screening Tool Based on the 5‐Question Geriatric Locomotive Function Scale for Locomotive Syndrome,” Journal of Orthopaedic Science 27 (2022): 913–920. 34090778 10.1016/j.jos.2021.05.001

[22] T. Kobayashi , T. Morimoto , C. Shimanoe , R. Ono , K. Otani , and M. Mawatari , “Development of a Tool for Screening the Severity of Locomotive Syndrome by the Loco‐Check,” Journal of Orthopaedic Science 27 (2022): 701–706, 10.1016/j.jos.2021.03.011. 33975750

[23] M. Taniguchi , T. Ikezoe , T. Tsuboyama , et al., “Prevalence and Physical Characteristics of Locomotive Syndrome Stages as Classified by the New Criteria 2020 in Older Japanese People: Results From the Nagahama Study,” BMC Geriatrics 21 (2021): 489, 10.1186/s12877-021-02440-2. 34503459 PMC8428127

[24] A. Seichi , A. Kimura , S. Konno , and S. Yabuki , “Epidemiologic Survey of Locomotive Syndrome in Japan,” Journal of Orthopaedic Science 21 (2016): 222–225, 10.1016/j.jos.2015.12.012. 26806332

