# Supplementary figures and images for: Differences in Item Discrimination of the 25‐Question Geriatric Locomotive Function Scale Between Younger and Middle‐Aged Adults and Older Adults: An Analysis Using the Item Response Theory
Source: Geriatr Gerontol Int. 2026 Feb 6;26(2):e70357. doi: 10.1111/ggi.70357 (PMC12879528; doi:10.1111/ggi.70357)

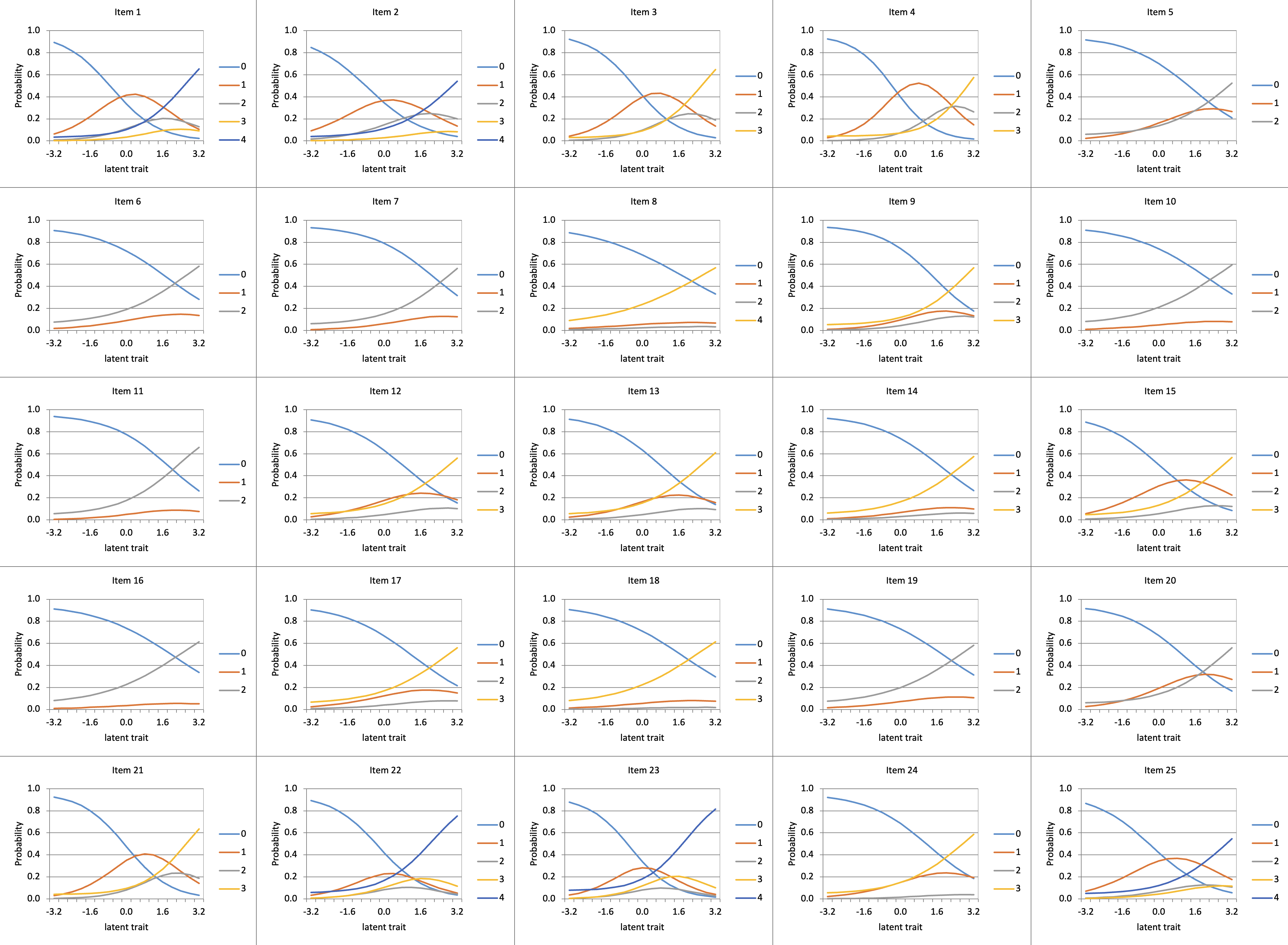

Supplement: Supplementary file 1 — Figure S1: Category characteristic curves for all 25 GLFS‐25 items, presented separately for younger and middle‐aged adults. [file GGI-26-0-s003.png]

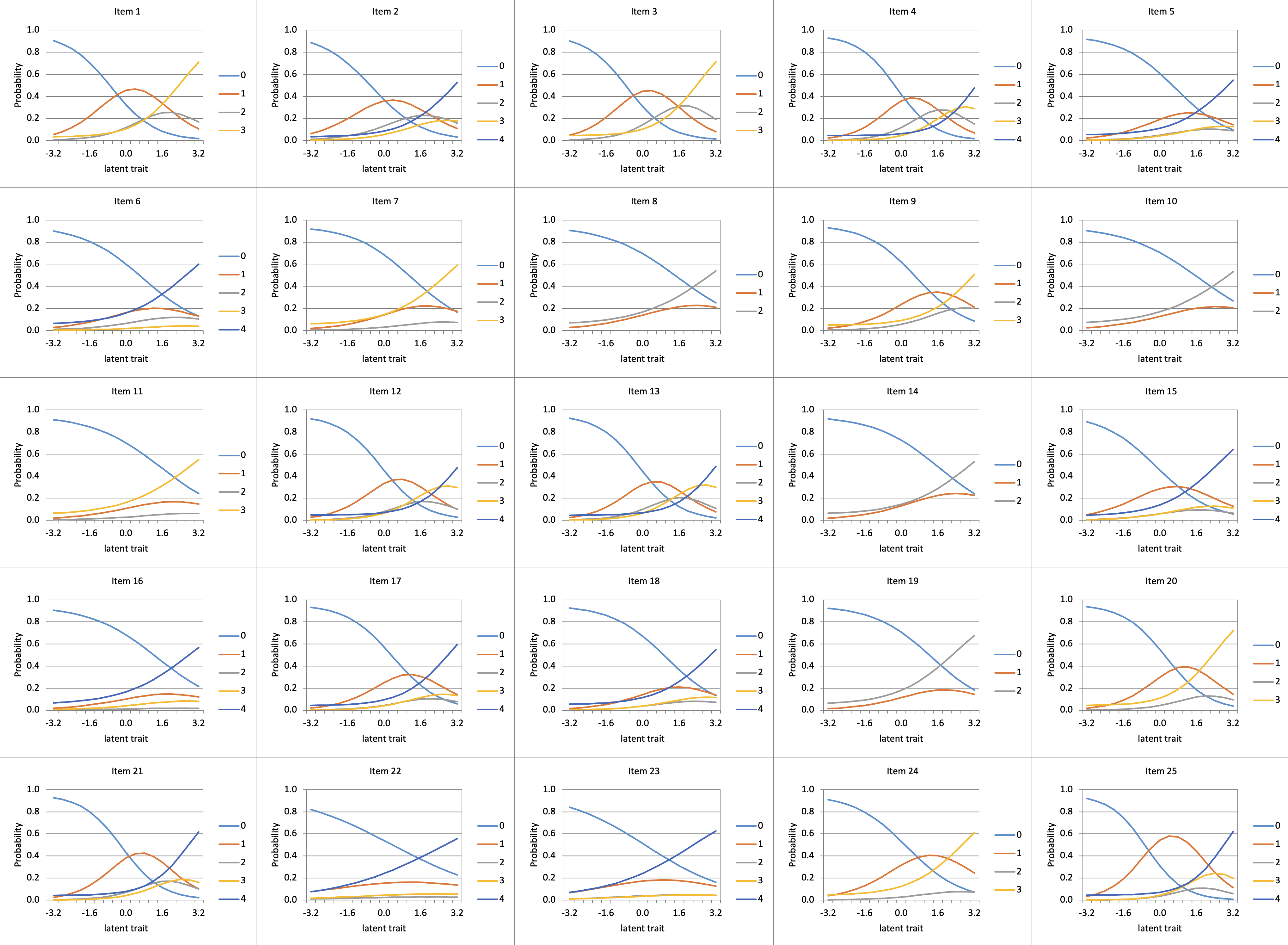

Supplement: Supplementary file 2 — Figure S2: Category characteristic curves for all 25 GLFS‐25 items, presented separately for older adults. [file GGI-26-0-s001.png]
