# Supplementary material for: Differences in Item Discrimination of the 25‐Question Geriatric Locomotive Function Scale Between Younger and Middle‐Aged Adults and Older Adults: An Analysis Using the Item Response Theory
Source: Geriatr Gerontol Int. 2026 Feb 6;26(2):e70357. doi: 10.1111/ggi.70357 (PMC12879528; doi:10.1111/ggi.70357)
Supplement: Supplementary file 3 — Table S1: Item‐level model fit indices for younger and middle‐aged and older adults. [file GGI-26-0-s002.docx]

**Supplementary Tables**

| Supplementary Table S1. Item-level model fit indices for younger and middle-aged and older adults | | |
| --- | --- | --- |
|  | Younger and middle-aged adults | Older adults. |
|  | RMSEA | RMSEA |
| Item1 | 0.000 | 0.037 |
| Item2 | 0.023 | 0.031 |
| Item3 | 0.065 | 0.027 |
| Item4 | 0.069 | 0.060 |
| Item5 | 0.025 | 0.035 |
| Item6 | 0.055 | 0.065 |
| Item7 | 0.023 | 0.078 |
| Item8 | 0.052 | 0.065 |
| Item9 | 0.074 | 0.032 |
| Item10 | 0.040 | 0.050 |
| Item11 | 0.000 | 0.056 |
| Item12 | 0.055 | 0.049 |
| Item13 | 0.080 | 0.071 |
| Item14 | 0.058 | 0.046 |
| Item15 | 0.043 | 0.052 |
| Item16 | 0.000 | 0.034 |
| Item17 | 0.040 | 0.080 |
| Item18 | 0.072 | 0.053 |
| Item19 | 0.000 | 0.044 |
| Item20 | 0.045 | 0.044 |
| Item21 | 0.068 | 0.078 |
| Item22 | 0.018 | 0.069 |
| Item23 | 0.000 | 0.088 |
| Item24 | 0.047 | 0.042 |
| Item25 | 0.079 | 0.040 |
| Item (overall) | 0.04 | 0.05 |
| Root mean square error of approximation (RMSEA) values are presented for each GLFS-25 item in younger and middle-aged adults (<65 years) and older adults (≥65 years). Lower RMSEA values indicate better model fit, with values <0.06 considered good and values <0.08 considered acceptable. Overall model-level RMSEA values were 0.04 for younger and middle-aged adults and 0.05 for older adults, indicating acceptable fit in both groups. | | |
